# Oncological Safety and Recurrence in the Surgical Treatment of Atypical Fibroxanthoma and Pleomorphic Dermal Sarcoma of the Scalp

**DOI:** 10.3390/cancers14122869

**Published:** 2022-06-10

**Authors:** Johannes Zeller, Jurij Kiefer, David Braig, Oscar Winninger, Daniel Kraus, Salome Hagelstein, Steffen U. Eisenhardt

**Affiliations:** 1 Department of Plastic and Hand Surgery, Medical Faculty of the University of Freiburg, University of Freiburg Medical Center, 79106 Freiburg, Germany; jurij.kiefer@uniklinik-freiburg.de (J.K.); david.braig@uniklinik-freiburg.de (D.B.); oscar.winninger@uniklinik-freiburg.de (O.W.); daniel.kraus@uniklinik-freiburg.de (D.K.); salomehagelstein@gmail.com (S.H.); steffen.eisenhardt@uniklinik-freiburg.de (S.U.E.); 2 Division of Hand, Plastic and Aesthetic Surgery, University Hospital, LMU Munich, 80336 Munich, Germany

**Keywords:** atypic fibroxanthoma, pleomorphic dermal sarcoma, dermal sarcoma, surgical treatment

## Abstract

**Simple Summary:**

Atypical fibroxanthoma and pleomorphic dermal sarcoma represent two tumors on the spectrum of a rare dermal sarcoma entity. Close clinical presentation and nearly identical histologic features but distinct prognoses make proper treatment strategies challenging. We performed a retrospective analysis of 32 patients with AFX or PDS in the scalp to provide guidance regarding the extent of radical excision to achieve stable oncological outcomes and whether radical tumor resection on the scalps required complex soft-tissue reconstruction. Compared to AFX, PDS shows a more aggressive growth pattern with frequent satellite metastases and distant metastases. These require extensive resections for local control to achieve long-term remission in most PDS patients. Despite the limited elasticity of the scalp, plastic reconstructive procedures can obtain reliable soft tissue reconstruction, even for complete scalp defects. Due to their rarity, managing these tumors requires an interdisciplinary setting in a specialized sarcoma center.

**Abstract:**

Atypical fibroxanthoma (AFX) and pleomorphic dermal sarcoma (PDS) are two distinct designations for a rare dermal sarcoma entity. These tumors arise predominantly in the sun-damaged skin of elderly patients. Although both AFX and PDS have a similar clinical presentation and nearly identical genetic features, they significantly differ in prognosis. Here we present a retrospective single-center chart review analyzing the outcomes of patients treated for dermal sarcoma. The radicality of the tumor-resection extent and soft-tissue reconstructive options were assessed. Patients between January 2010 and August 2021 were included. We recorded resection margins, tumor recurrence, overall survival, number of operations until complete tumor resection, and reconstructive procedures; any complications were recorded. Furthermore, we analyzed a subgroup of patients with satellite metastases. A total of 32 patients met the inclusion criteria (30 male, 2 female, median age of 77.5 years (interquartile range (IQR) 74–81)). Histopathology revealed AFX in 14 patients and PDS in 18 patients. Margin-free resection was achieved in 31 cases, and 27 patients were remission free over the reported period. The local recurrence rate was 5, and distant metastasis was detected in four cases. Of all the PDS cases, nine presented with satellite metastasis. No AFX had satellite metastases. Due to their rarity, managing these tumors requires an interdisciplinary setting in a specialized sarcoma center.

## 1. Introduction

Atypical fibroxanthoma (AFX) and pleomorphic dermal sarcoma (PDS) are two distinct designations for a rare sarcoma entity of fibro histiocytic mesenchymal origin currently distinguished mainly using histopathological criteria [1,2,3]. Compared to the AFX, PDS, as a manifestation of this entity, displays more aggressive clinical behavior. It presents with an invasion of deeper tissue structures, lymph vessels, and nerves [4] and is categorized as a tumor with low-grade malignant potential based on its potential for local recurrence and metastasis [5]. The AFX is considered the non-infiltrating precursor lesion of PDS, and both are considered a disease spectrum rather than two distinct entities [6,7].

However, the exact relationship between AFX and PDS has been debated for many years. Only recently, multiple genetic studies have supported the concept of AFX and PDS comprising a single entity. Griewank et al. contributed to a better understanding of these tumors’ genetics. Both tumors present a similar gene alteration profile with frequent mutations in NOTCH1/2 and FAT1, TP53, and the TERT promoter [4,8].

In line, Koelsche et al. reported that copy number profiling revealed alterations with a similar frequency and distribution, with deletions most frequent in 9p and 13q, and DNA-methylation profiling utilized did not separate AFX from PDS [9]. Yet, both AFX and PDS lack the expression of lineage-specific markers. Therefore, the diagnosis of this group of ambiguous definitions is still made by excluding other neoplasms with similar morphology [10]. Given the high frequency of mutations with a UV signature in TP53, UV-signature mutation analysis supports UV exposure being a relevant pathogenic event in both AFX and PDS, respectively [4]. Further, the clinical presentation of both tumors endorses the concept of UV exposure as a significant risk factor, as follows: both tumors frequently arise in elderly, predominantly male patients, and heavily sun-exposed areas, i.e., the scalp and head with less or no hair protection due to a receding hairline in males.

Nevertheless, the AFX and PDS have strongly differing prognoses, and both the similar clinical presentation and the genetic features in histology impede the discrimination vital for suitable treatment. Therefore, interdisciplinary treatment, consisting of surgical treatment and (neo-)-adjuvant radiotherapy in selected cases, is the mainstay of the curative therapy for AFX and PDS [11]. While AFX has a very low local recurrence rate and only single cases of metastasis are reported, PDS features significantly more aggressive behavior with a potentially life-threatening clinical course [5]. The scalp’s rigid and complex anatomy may further complicate adequate treatment as follows: tumor salvage and excessive soft-tissue defects may oppose the feasibility of soft-tissue reconstruction [12]. These conflicting interests leave the team handling these cases questioning how radical the resection should be to ensure oncological safety.

Here, we present a single-center retrospective analysis of 32 patients with AFX or PDS on the scalp. We analyzed the two neoplasms regarding the extent of radical excision to achieve stable oncological outcomes. We further assessed whether radical tumor resection on the scalps required complex soft-tissue reconstruction. Our findings will provide guidance in identifying the subgroup of patients that potentially benefit from radical excisions and microsurgical reconstruction.

## 2. Materials and Methods

### 2.1. The Patients and Methods

A retrospective chart review was conducted to identify and analyze data concerning all patients that underwent scalp reconstruction with local and free flaps after AFX or PDS resection at the Department of Plastic and Hand Surgery Freiburg between January 2010 and August 2021. All patients received appropriate tumor staging diagnostics, followed by an interdisciplinary tumor board presentation.

Area and depth of excision defects were obtained from surgical reports and standardized patients’ photographs. Histopathological reports were reviewed for resections margins and depth of infiltration. Excision defects were reconstructed primarily or after temporary coverage. Epidemiologic data included the patient’s age at the time of surgery, gender, and comorbidities were noted. We classified the physical status of each patient at the time of the operation according to the American Society of Anesthesiologists classification (ASA) [13]. Ethical consent was obtained from the local ethics committee (434/19).

### 2.2. Statistical Analysis

Statistical analysis was performed using GraphPad Prism v9 for Mac, GraphPad Software, La Jolla, CA, USA. Normal (Gaussian) distribution was tested and rejected for margin clearance, defect sizes comparison (AFX and PDS), and recurrence rate. Moreover, an unpaired Mann-Whitney test with a two-tailed *p*-value was performed. Unpaired *t*-test (Student’s test) was used for parametric statistical analysis. A *p*-value < 0.05 was considered statistically significant.

## 3. Results

### 3.1. Patient Characteristics

Thirty-two patients met the inclusion criteria, with 30 male and two female patients. The median age of all patients was 78 (IQR 72–81) years. Patients presented with a mean ASA score of 2.55 ± 0.61. Most frequently, comorbidities included coronary heart disease, hypertension, atrial fibrillation, and obesity as assessed by their body mass index [14,15]. These were the encountered comorbidities. One patient suffered from a chronic form of graft-versus-host disease (GVHD) with dermatologic manifestations after lymphoma treatment and allogenic bone marrow transplantation.

### 3.2. Surgical Procedures

Patients were assigned to our clinic by the interdisciplinary tumor board after external operations in 87.5% of all cases; 12.5% of all cases presented primarily to our clinic. This study included all patients with a pathologically confirmed diagnosis of AFX or PDS localized in the scalp. Biopsies and pathological reports were assessable in every case.

When the resection status or definitive pathological report was uncertain, we performed a temporary wound dressing with a synthetic skin replacement, EpiGARD^®^ (BioVision, Inc., Oslo, Norway) in eight cases (*n* = 6 for AFX and *n* = 2 for PDS). Temporary closure occurred in small and mid-area defects (13.05–31.16 cm^2^, median defect size 20.52 cm^2^), and wound closure was performed after obtaining tumor-free margins. All patients in this subgroup had previous resections with positive margins performed in other hospitals.

### 3.3. Defect Sizes

**Figure 1:**
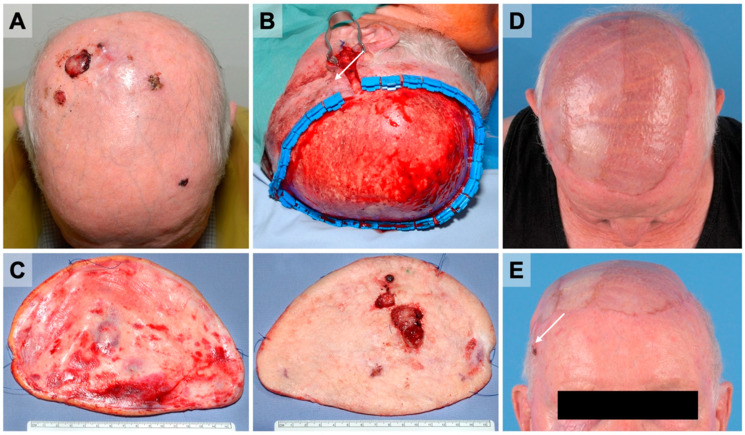
An 81-year-old male patient presented with an external diagnosis of PDS after failed excision biopsy (**A**). (**B**) After wide resection, a full-thickness scalp defect of 221 cm^2^ was closed using a latissimus dorsi flap and STSG. (**C**) The resected scalp showed wide tumor-free resections margins (2 cm) and no infiltration of the bone. (**D**) The three-month postoperative control showed satisfying aesthetic results. (**E**) However, a new lesion near the anastomosis had occurred (arrow). The second resection was performed with microscopically positive resection margins. Due to its close proximity to the flap, anastomosis adjuvant radiation was favored and conducted.

Total defect sizes ranged from 3 to 288 cm^2^ with a median of 27.2 cm^2^ (IQR 12.6–37.5). When comparing the AFX and PDS subgroups, we found no significant difference in median defect size (16.0 cm^2^ (IQR 13.1–35.1) for AFX and 31.3 cm^2^ (IQR 8.8–44.2) for PDS, respectively, *p* = 0.406). However, the median defect size in the subgroup of patients with satellite metastases was 238.8 cm^2^ (IQR 93.4–280.1). A typical case of satellite metastases and large defect size is presented in Figure 1.

### 3.4. Resection and Clearance Margins

The pathology reports were analyzed for macroscopic clearance margins and tumor-free resection margins. Tumor-free margins were achieved in 84.4% after the first operation in our department, and ultimately in 96.9% of cases after a mean of 1.05 operations. In cases of tumor recurrence, the counting started again, and biopsies, were not taken into account. The deep margin showed infiltration of the periosteum in 2 patients. Whole-thickness skull resection was not performed in any of the reported cases.

In multifocal or satellite tumor resection, clearance margins were disproportionate and significantly wider at 4.2 cm (IQR 3.55–5.05; *p* < 0.0001). However, with both being total scalp resections, the mean of the deep resection margin did not differ significantly (0.2 ± 0 cm vs. 0.28 ± 0.18 cm, *p* > 0.05). None of the tumors with positive resection margins was an atypical fibroxanthoma (margin clearance 1.50 cm at the median (IQR 1.4–2)).

Based on the concept that AFX and PDS are part of a disease spectrum rather than two distinct entities [6,7] and that AFX is considered the non-infiltrating precursor lesion, we analyzed cases of PDS with an initial diagnosis of AFX. However, all PDS reported in this study presented initially with PDS and no AFX differentiated to PDS, respectively.

### 3.5. Reconstructive Procedures

**Figure 2:**
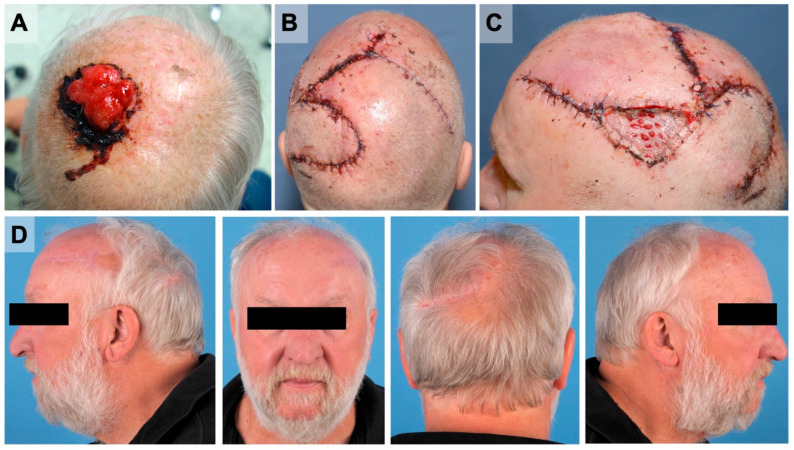
Reconstruction after AFX resection using the Orticochea flap and STSG was aesthetically satisfactory. (**A**) A 63-year-old patient presented with an AFX with exophytic growth in the parietal scalp closely left to the median line. (**B**) An Orticochea flap design (“banana peel flap”) was planned and conducted to close the 43.56 cm^2^ large defect after R0 tumor resection. (**C**) STSG from the patient’s upper back was used additionally for wound closure. (**D**) The three-month control shows a tumor-free and delighted patient. The hair borders were considered during the planning of the flaps and resulted in a normal hairline.

**Figure 3:**
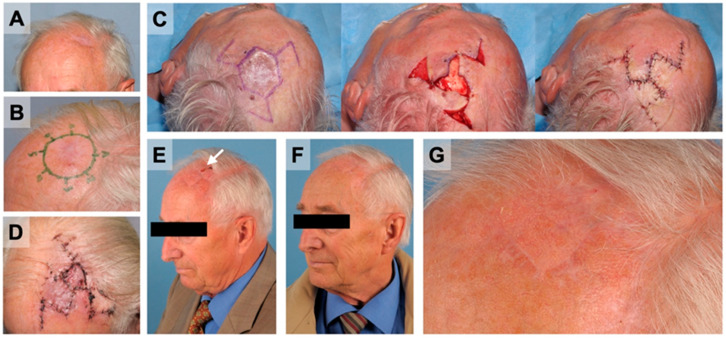
Triple-rhomboid flap for the reconstruction of the scalp in the frontotemporal region. (**A**) A 77-year-old male patient presented after resection of an AFX in an external hospital with positive margins in the frontotemporal area. (**B**) The patient was treated in a multistage approach, with temporary coverage to ensure tumor-free margins. The follow-up resection showed no residues of tumor tissue. (**C**,**D**) The resulting full-thickness scalp defect (middle) of 13.05 cm^2^ was closed using a triple-rhomboid flap. (**E**) One month after the definitive resection, the patient presented with minimal wound dehiscence (arrow). (**F**,**G**) The three-month postoperative control showed satisfying aesthetic results without clinical signs of local recurrence.

**Figure 4:**
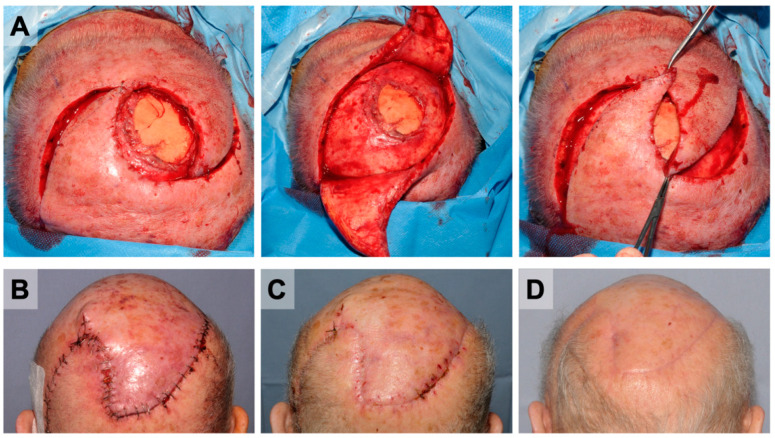
Yin-Yang flap for the scalp reconstruction after margin-free resection of an AFX. Presentation of a 78-year-old patient with AFX in the mid-scalp region in transition to the vertex. The tumor was resected, including the periosteum, in a first operation and temporally covered with synthetic skin replacement EpiGARD^®^. (**A**) Within four days, tumor-free margins were confirmed, and coverage of the 29.5 cm^2^ was performed by yin-yang rotation flap. The later course was uneventful. (**B**) The results on postoperative day 3, (**C**) one month after the surgery, (**D**) and at the 3-month control show the patient presenting with good wound closure and without complications or recurrence.

Definitive reconstruction was achieved with primary closure (*n* = 4), local transposition (*n* = 7) and rotation flaps (*n* = 12), and one pedicled temporal fascia flap with STSG (*n* = 1), full-thickness skin graft (*n* = 2), and free flaps (*n* = 8), respectively. Local flaps consisted of Orticochea (Figure 2), rhomboid and triple-rhomboid flaps (Figure 3), and yin-yang flaps (Figure 4). For patients requiring free flap coverage, we performed fasciocutaneous anterolateral thigh (*n* = 3) and latissimus dorsi flaps (*n* = 5). Temporary wound dressing with synthetic skin replacement was performed in eight cases.

### 3.6. Adjuvant Therapy

Adjuvant radiotherapy was performed on six patients, all of whom were diagnosed with PDS. The inclusion of adjuvant radiotherapy into the treatment plan was made individually and in an interdisciplinary tumor board setting, consisting of oncology, oncological, reconstructive surgery, and radiotherapy experts. (Neo)adjuvant radiotherapy was performed in no case of AFX. One of the six patients receiving adjuvant radiation was diagnosed with incomplete tumor resection. The subgroup of PDS patients diagnosed with satellite metastasis consisted of five patients, with one patient having microscopically incomplete resections.

### 3.7. Recurrence Rate

All patients with dermal sarcomas received regular clinical follow-ups every 3–6 months (PDS, AFX) after complete resection for the first 2 years. Then, the follow-up interval was extended to 6–12 months for another 3 years. Afterward, most patients received regular prevention by their general practitioner or dermatologist. Patients had follow-ups for at least five years (60 months). During this period, no patient within the AFX group presented with a local recurrence or tumor metastases.

The local recurrence rate in the PDS group was 5 patients (27.8%). The earliest recurrence within the PDS group was after one month in two cases, both with the initial presentation of satellite metastases. The subgroup of PDS patients with satellite metastases showed significantly shorter recurrence-free periods (median 10 months, IQR 3.25–18.5) than patients with unifocal PDS, which showed only local recurrence in one case after 10 months (median 60 months, IQR 60–60, *p* < 0.0001).

### 3.8. Survival

Over the period of clinical follow-up, three patients died of disease-specific deaths (all PDS). All three patients showed distant tumor recurrence (lung metastases). The survival was 24 months (median) after primary resection.

### 3.9. Complications

Overall, peri-operative complications in the analyzed group of patients were uncommon, with two major (partial flap loss; intraoperative iatrogenic nerve injury without functional recovery) and two minor complications (an intraoperative blood transfusion in one case and prolonged wound healing in another one), respectively. In one patient, the frontal branch of the facial nerve was resected due to its proximity to a PDS. The defect was covered with a rhomboid flap, and, in addition, a direct brow lift was performed. The second major complication was sepsis requiring ICU treatment and a partial flap loss in one patient treated with a free latissimus dorsi flap and STSG. The patient suffered from a chronic form of GVHD. Afterward, both patients showed an uneventful course.

## 4. Discussion

The head and neck area comprises 4–10% of all newly diagnosed soft tissue sarcomas [16]; however, 90% of AFX manifestations occur here [17]. Multiple studies found the scalp to be the most common region for PDS [5,18,19]. UV exposure and irradiation are known risk factors for developing AFX and PDS. Therefore, the primary group of patients is older men with an occurrence in the scalp. In line with that, the cohort analyzed in this study included 30 male and two female patients with a median age of 77.5 years (IQR 74–81). Although patients are usually seen at an early stage of the disease due to their prominent location, these tumors often present at substantial sizes due to their rapid growth [20]. The lesions are usually present as a solitary plaque or nodule, commonly with central ulcerations (Figure 1 and Figure 2) [21]. Patients often undergo initial surgery at a small volume center not experienced in treating dermal sarcomas, which substantially increases the oncologic and reconstructive challenge. Nearly all patients reported as a result of this were previously operated on with microscopically positive margins before being referred to our department, which is in line with previously reported data [22].

The treatment plan for the presented cases of AFX and PDS in the scalp consisted of an initial biopsy and staging imaging, followed by an interdisciplinary tumor board decision. The resection was planned based on the clinical appearance, comorbidities, imaging, and pathology reports. The ultimate goal, however, must be clear margins [23], as incomplete initial resection causes local recurrence [24,25].

In the past, excision with 2 cm margins was recommended for both entities. However, to date, no guidelines for surgical treatment and follow-up have been established [23]. A recently published probability model based on a systemic review by Jibbe et al. found peripheral excision margins of at least 2 cm for AFX and 3 cm for PDS, respectively, to reduce the recurrence rate [26]. However, the current standard of care in the treatment of AFX is due to its intermediate malignant potential, micrographically controlled excision with a narrow clearance margin appears to be sufficient [11]. In any case, margin-controlled surgery is favorable for ensuring complete removal and reducing resection morbidity [26]. Although the number of cases is limited, we found no local recurrence of atypical fibroxanthoma when margin-controlled surgery was performed with a margin clearance of 1.50 cm at the median (IQR 1.4–2).

There is only limited evidence that neoadjuvant radiotherapy for the treatment of PDS on the scalp is effective [18,27,28]. Within the reported cases, we found radiotherapy primarily as an adjunctive treatment. In one case, microscopically incomplete resections, the discission for adjuvant radiotherapy was made due to the proximity of the local tumor recurrence to the anastomosis of the flap reconstruction (Figure 1B,E) and to prevent the imminent risk of injury to the free flap pedicle. This patient, later on, developed metastasis. Recent studies have reported that radiotherapy should not be used in cases of incomplete resection margins as a treatment modality to bypass large defects [23]. For patients treated at our department, the decision for adjuvant radiotherapy was made based on the interdisciplinary tumor board decision. All patients presented disseminated growth and deep margins, close or in direct contact with the external tabula. However, our study’s limited number of patients does not allow for a definite statement regarding radiotherapy in patients presenting with PDS.

In line with previously published data, we report no recurrence of AFX nor distant metastasis in any patient after complete excision [7,29,30]. The recurrence rates of PDS on the scalp were previously described by a meta-analysis of nine studies as 23.8%, with a local recurrence of 19.92% and metastasis in 7.14% of all cases over a mean follow-up of 13–46.4 months [31]. The analyzed subgroup of patients with satellite metastasis of PDS in this study aligns with this data.

Aesthetically and functionally, the treatment of AFX and PDS after wide resection of the scalp proves to be a challenging entity for reconstruction. Further, the scalp itself represents an anatomically complex area with minimal intrinsic laxity and critical structures as the brain is nearby. Thus, the pathological discrimination of AFX and PDS is crucial due to their clinically distinct tendency for local and distal tumor recurrence [5,24].

To provide oncological clearance margins in PDS, wide resection is favorable due to its tendency to develop satellite metastasis and local recurrence; however, this aggressive approach may also lead to an increased demand for complex soft-tissue reconstructions, e.g., free tissue transfer, in this group.

AFX and PDS pose a diagnostic challenge with conventional analytic methods [9]. However, due to the lack of a specific tumor marker, the histopathological discrimination of PDS from AFX depends on the invasion of deeper tissue structures (subcutaneous involvement), necrosis, and lymphovascular and perineural invasion [9].

The cohort of patients suffering from rare sarcoma entities can be highly complex and demanding [32,33]. In the analyzed group complicating cardiovascular disorders and medication were present in every patient. Due to the need for an interdisciplinary treatment and the rarity, patients with AFX and PDS should be referred and treated exclusively in high-volume cancer centers. The metastatic potential of PDS and the high probability of local recurrence emphasize the need for a pathology department capable of exact diagnosis and a proficient surgical team to ensure complete resection of the tumor in the treatment of PDS of the scalp.

The radical surgical resection and sufficient scalp reconstruction represent the best treatment options to cure malignant tumors in this anatomically critical region. Overall, the soft-tissue reconstruction of scalp defects after oncological resection has proven to be highly demanding in patients with AFX and PDS, respectively. The scalp can be shaped to meet most requirements; however, the major limitation is its rigidity, which can be addressed by scoring local flaps or using a free flap.

The presented study has several limitations that should be considered when interpreting the findings. Due to the rarity of both entities, the overall number of patients in this retrospective analysis is relatively low. Thus, the character of the study is more descriptive, reporting our experience and treatment algorithms for dermal sarcomas. Furthermore, the retrospective analysis represents a single-center experience over a 10-year period with several surgeons involved, which restricts the universal applicability of the results. Second, the number of AFX and PDS patients included in our study is relatively small. Ideally, a multi-center study can be implemented. Third, due to the character of the study, the results given in this study, including more patients, are needed to further improve treatment strategies and optimize follow-ups of these rare dermal sarcomas of the scalp.

## 5. Conclusions

AFX and PDS of the scalp present a challenge for the pathological assessment and the oncosurgical treatment likewise. Both entities are histopathologically and clinically challenging. Advances in reconstructive (micro)surgery, even after excessive scalp defects, can give stable oncological outcomes with satisfactory aesthetic results, and preservation of the soft-tissue continuity can be achieved. As reported before, radical surgery to achieve initial tumor-free margins, followed by individualized reconstruction, should be the goal of treatment, while the role of radiotherapy stays elusive. However, to further improve the treatment of this rare entity of dermal sarcomas, the demand for a large-sized prospective study is given.

## Data Availability

The datasets analyzed in this manuscript are not publicly available. Requests to access the datasets should be directed to johannes.zeller@uniklinik-freiburg.de.
